# Odonto-Chirurgical Society

**Published:** 1886-03

**Authors:** 


THE
### AMERICAN JOURNAL
OF
### DENTAL SCIENCE.

VOL. XIX. THIRD SERIES.— MARCH 1886. NO. 11.

ARTICLE I.

### ODONTO-CHIRURGICAL SOCIETY.

At the December meeting of this Society, which took place in the Society's Rooms, Edinburgh, Mr. W. Bowman Macleod, President, in the chair, the discussion upon Dr. H. H. Edwards' paper, on the "Missing Incisors in Man" was opened by Mr. Wilson, who said he had listened to the reading of the paper with much pleasure, and he was very much pleased to learn that the short paper he had read to the Society in March had had the effect of bringing into the field such a close observer and skillful artist as Dr. Edwards.

Case 1 shows a state of the incisors he had never seen in the pernament series, and but twice in the temporary. He differed from Dr. Edwards in that he considered the extra teeth to be not the central pair, but the first lateral, although these are remarkable as being larger than the central; one thing certain was that it was not the second, or outermost lateral pair.

Cases 2 and 3 represent cases with which most present were familiar.

In Case 4 he did not see any ground for calling the incisor in relation to the right canine anything but a dwarfed normal lateral, difference of form in the pair of these teeth being not unfrequent, and the space between it and the central was not so large as to require accounting for.

In Case 5 the space occupies the line of the maxillo-premaxillary suture, and it is more likely to be connected with the development of the bones than with the teeth.

Case 6 was, in his experience, a common form when the laterals were suppressed.

In Case 7 the incisor between central and canine on the right side he regarded as a dwarfed normal lateral, and case 9 only differed from it in that the lateral is more rudimentary in form. In the majority of similar cases which had come under his own notice, the solitary lateral was conoid.

Case 8. The tooth so beautifully reproduced in ivory he did not consider a germinated tooth at all, but what was much rarer, a double-rooted lateral, the extra root being due to the malformation of the cingulum, the two sides of which had not united, the small root being a continuation of the distal half.

Laterals were extremly liable to have the cingulum malformed, but it was certainly very rare to find it resulting in an extra root, as in this case.

He had only met with one, which he would pass round, and they would see that it only differed from Dr. Edwards' one in that the distal root continued in contact with the larger root.

As showing that the same thing occurred in other teeth he passed round an under canine, in which they would see that a fault in the labial cervical margin of the enamel was accompanied by the presence of an extra root, which started just a little below the neck on the same surface.

As regarded the use of the term "geminous tooth," or as more frequently styled geminated, Dr Edwards was quite correct, as there are three forms—1st, lateral union of both crowns and roots; 2nd, union of crowns, roots more or less distinct; and 3rd, crowns more or less divided, roots united.

The existence of one or more pulp cavities and canals, depending on the extent to which the conjoined teeth had lost their individuality.

The quotation from Dr. Thomson's paper, however well it might apply to members liable to hypertrophy or atrophy, according as the individual used them, bore little on the subject before them. Hereditary defective structure was extremely general, but certainly not any prevalence of aberrant, or rudimentary forms. Conoid laterals, biscuspids, and third molars are still in a small minority.

It might be said that want of use leads to defective development of the jaws, and that in turn to suppression of certain teeth owing to want of room, but this conclusion is largely an assumption.

Undoubtedly rudimentary forms in certain teeth, as also suppression of the same, are hereditary more or less for a generation or two, but the customs of civilized man do not encourage their persistence. In the lower animals either could readily be made a pernament variety.

As to the suppressed incisor being the central one, he thought that on two grounds they might be put out of court —1st, the incisors in man being largely prehensile, and the centrals normally the strongest and most important, they should be the last to be suppressed; 2nd, when five or six incisors are present, it is very exceptional for more than two (the centrals) to be of the central type.

No conclusion can be drawn from the period of eruption as in the permanent series the overcrowding frequently leads to the retarded eruption of the centrals long after the laterals and supernumerary teeth were in place. In two cases of extra incisors which came under his own observation, the middle or second incisor erupted after both the central and outer incisor were in place. While in the third all four laterals were more or less advanced (the outer ones most so) before the centrals erupted.

In the temporary series there was little irregularity produced by their presence. but there seems a strong tendency to the extra incisor being geminated to one or other of the normal ones.

What he regarded as the typical form of the lateral incisor was just that described by Dr. Edwards. It differs from the central (taking its labial aspect only) in being more V-shaped, in its lateral convexity being greater and most pronounced nearer to its mesial side, and in being shortened to and rounded off at its distal cutting edge.

Digressing to the missing premolars, his own observations would lead him to say they were the third and fourth.

As regarded the supernumerary teeth not unfrequently met with to the buccal surface of the molars, he was rather puzzled, having met them between the first and second, second and third, and in one case to the distal side of the third molar. He had also met with a few cases in which they were geminated with one or other of the molars So far as he had seen they differed considerably in form from those met with in the front of the nouth.

He was afraid he had occupied too much of their time, and would conclude by asking them to compare closely the lateral and canine he had sent round with the lateral (Case 8) of Dr. Edwards', and say whether they agreed with him.

Mr. Amoore said he had listened with great interest to Mr. Wilson's close criticisms upon the paper, and if there was more to be learned from differences of opinion than concurrences, they might learn something from what they had heard that evening. He dissented from Dr. Edwards in his opinion that, of the incisor teeth, the centrals would be the most likely to be suppressed first. To quote from the text of his paper—"The incisors are the teeth of prehension, and the centrals naturally are the most prehensile; therefore, if suppression has taken place through disuse, I infer that the original centrals would be the first to disappear." Now, if suppression has taken place, it would surely be the less used side teeth which would disappear previous to their more serviceable central neighbors. This view is borne out by references to comparative dental anatomy, where the side teeth sometimes become quite rudimentary or are lost, while the centrals often rather developed and increased in size and strength.

He then exhibited a model of a well-formed upper jaw in which there were two teeth erupted buccally between the second and third molars, and which tallied exactly with a similar case referred to and described by Dr. Edwards. One of the supernumeraries had been extracted and showed a conical root, while the crown more resembled a bicuspid than a molar, and although he had no strong grounds for his opinion, he could scarcely bring himself to believe that they were representative of a lost third pre-molar, springing up in this out-of-the-way position; he thought it rather straining a point, whenever a supernumerary tooth appeared, to give to it a place as a reappearance, in imperfect form, of a tooth once commonly present but now suppressed. In the same way he differed from Dr. Edwards, though confessing that he had not given the matter so much attention as he (Dr. Edwards) had done, in so often assigning, as a reason for spaced teeth, that it was an effort on the part of Nature to allow room for a tooth once present in the jaw, but now absent. This was notably the instance in Case 5, already referred to by Mr. Wilson, in which a space existed between lateral and canine on the one side of the mouth only, and, as had been remarked, was more likely to have been caused by an irregularity in the development of the bone at the inter-maxillary suture—or possibly the undue retention of a temporary tooth on that side, or it might even be due to an irregular articulation with the lower jaw. How common it was to find, when the laterals were absent, that the centrals were spaced, and when, during of eruption of the incisor teeth in children, a long interval of time elapsed between the appearances of the centrals and laterals; the centrals often remained with wide spaces between them until the laterals came down, when the space gradually closed up. He had models of a jaw in his possession which he should have liked to have had Dr. Edwards' opinion upon; it was a typically well-formed and faultless mouth and jaw, with the teeth all present, but on either side between the canines and the first bicuspid there existed well marked spaces. From their position he would attach no significance to the circumstance, attributing it rather to the effect of articulation with the lower jaw than to any other cause. Returning, however, to the missing incisor again, he had a case, of which, he regretted, he was unable to exhibit the models. In it a temporary supernumerary lateral was succeeded by a supernumerary permanent lateral, in either case it being impossible to determine, from the crowns, at all events, which was the extra tooth.

With regard to the carved ivory model of a lateral tooth, which they all admired so much, he was more inclined to agree with Mr. Wilson's views upon the question, and think it more probably a double-rooted lateral than a geminated tooth, although he scarcely thought that the teeth Mr. Wilson had passed around proved his point, though they certainly tended to confirm it.

He trusted that any exceptions that he himself, among others, had taken to Dr. Edwards' paper, would not be taken as due to a want of appreciation, but when, as in the present instance, there were so very few reliable data to argue from, there would necessarily exist many differences of opinion.

After several members had taken part in the discussion, the President said that nobody would be more pleased than Dr. Edwards himself in reading those opinions which differed from his own opinions. The whole of the paper was more or less a tentative one, expressing views which might reasonably be deduced from facts accompanying it, but by no means claiming for these views the value of demonstrated deductions. The great object Dr. Edwards seemed to have in his paper was to contribute a few more facts bearing upon an interesting and much neglected vein of research, in order that interest in it might be further awakened and perpetuated in this direction, and tend to the collection of such a mass of instances and illustrations as would furnish a sufficiently broad basis upon which to build a conclusion. The paper had most admirably fulfilled its purpose, and he hoped that they would be frequently favored with communications from their youngest corresponding member.

The President then called on Mr. Watson for his promised demonstrations for the evening,

Mr. Watson proceeded to give an exhibiton of slides, illustrative of Dental Pathology and Physiology, by means of the Limelight Lantern Microscope.

Without attempting to describe the instrnment technically; there were a few points in connection with it that would be interesting to remark upon. To fit up the lantern for the microscope, the ordinary objective in connection with it is removed, and the microscope lenses and apparatus screwed on, the objectives used on this occasion being 150, 90 and 30 diameters, on the relative value of which, in connection with the lantern, some remarks are made further on. One of the most ingenious contrivances in connection with it was the method by which a continual supply of oxygen could be maintained without the inconvenience usually attendant when bags are employed. The gas-holder consists of a bell capable of containing about 1½ cubic feet of gas, inverted in a water chamber below, holding a good sixed bucket full of water. This arrangement when in use serves as a stand for the lantern, and when emptied for travelling, the lantern can be packed away within it. The supply of oxygen is obtained from heating by means of a Bunsen burner, a cake made of powdered chlorate of potash, and the black oxide of manganese enclosed in a strong iron retort, whence the oxygen is evolved and conducted by a tube into the gas-holder; when more gas is required, another cake is supplied, and the process repeated, and thus a persistent supply of the gas can be maintained as long as desired.

The exhibition was a very interesting one, and showed the microscopical conditions of the teeth, from the germ to maturity, and in the different stages of disease; and toward the end sections of tumors having relation to the mouth were also exhibited. The sections were best seen with the lower powers of the microscope, as at present it was impossible with the instrument to bring out the smaller details as clearly as when the object was examined upon the stage of the microscope in the ordinary way, the definitions becoming indistinct as the powers were increased.

Later on, Mr. Watson exhibited some photo-micrographs of the dental structures, in which the details were brought out in a very satisfactory manner, and if by further adjustment and development the lantern microscope could be made to transmit as clearly the image, from the mounted specimen to the canvas direct, it would prove an almost indispensable instrument for purposes of instruction in class lectures.

At the conclusion of the exhibition, the President complimented Mr. Watson on the success of the demonstration, and tendered the thanks of the Society to Mr. Watson for his interesting and instructive exposition of the microscopic structures of the teeth.

On January 14, the third General Meeting of the Session was held, the President, Mr. Bowman Macleod, in the chair.

Mr. Price was called on for his remarks on Dr. Coffin's method for treating irregularities of the "split plate" and the use of piano wire. Through the kindness of Mr. Harold Coffin, he was enabled to exhibit a large series of models, illustrating the different types of results that could be obtained through its agency; and also plates and cases in various stages of preparation, showing the most advised methods of construction. As the subject had been so thoroughly treated by Mr. Walter Coffin, and already chronicled in the Transactions of the International Medical Congress in 1881, (vol. III p. 542; also in *Dental Record*, vol. I p. 112), it was thought unnecessary to republish any of the matter in the Society's Transactions, but as many of those present had either not seen, or had no opportunity of examining the models at leisure they were the occasion of considerable comment, and some of the details of their construction and technical "wrinkles" were new to several. Many of the members who had adopted it testified to its simplicity and value, at the same time venturing to doubt if, in some of the examples before them, the expansion treatment had not been ill advised, and that extraction would have yielded better results; but as they had not the lower articulations with them, nor the contour of the patient's face to assist them in their judgments, they were unable to pronounce any final opinion.

In summing up the opinions of members, the President said that he regretted very much the impossibility of sharing with absent members the great good which those present derived from the presentation of such a practical subject in the form in which it had been brought before them. Most of the members questioned the expediency of some of the operations for expansion of the arch as illustrated by the models on the table, and he quite agreed with them, and very possibly Mr. Coffin might now also share their views. One thing, however, was certain, that the introduction by Mr. Coffin of piano wire for regulation of teeth and expansion of the arch was a decided step in advance of any previous contrivance for these purposes. It was a remarkable illustration of the power of simple instruments when directed by brains. He had much pleasure in moving a cordial vote of thanks to Mr. Coffin for the loan of the series of illustrations, and also to Mr. Price for his most entertaining and lucid description of them.

### COMMUNICATIONS.

A casual communication from Mr. John Wood, Dumfries, was read, referring to the untoward accident in Mr. Saunders' practice at Barnstaple, in which the blade of a pair of upper biscupid forceps broke off during an operation, and slipping down the trachea, became lodged in the right bronchus, from which it was extracted by Sir William M'Cormac, seven weeks afterwards. It appears that the broken instrument has the name of "Evrard" upon it, but some doubt has been expressed as to whether it is a genuine instrument of his make, or merely an imitation of a spurious character. Be that as it may, by way of showing that such an accident is possible, even where there can be no question as to the genuineness of the manufacture, I summit for inspection by the members of the Society, a pair of excising forceps by Evrard, in which there is a fracture extending half way across the neck of the left blade in pretty much the same situation, and so far in the same direction as that which the line of fracture takes in the broken pair figured in the *Lancet* and the *Journal of the British Dental Association*. As will be observed, however, the fracture in this pair, instead of continuing its course *across*, has taken an inclination towards the line of the handles, which in some measure accounts for the blade not breaking off altogether, as in the Barnstaple case. That this instrument was made by Evrard is beyond dispute. It bears the well-known impress of his name, and forms one of a set of twenty instruments furnished by the late Mr. Evrard to special order, and presented to me by a patient. The mishap to this pair happened a short time ago, whilst being used in excising an upper front tooth, and surprised me not a little, having regard to the apparent strength of the part at which they gave way.

Mr. Stirling showed a continuous gum facing, from Verrier's furnace, where the platinum wire was soldered to the pins of the teeth with dental alloy. He said the advantage of dental alloy over pure gold as a solder is that it remains around the pins (where it is placed) until after the piece has been finally fired, whereas the gold usually runs away from the pins and flows all over the platinum wire.

Mr. Stirling also presented to the Society's museum the molar of a horse, the roots of which were involved in an odontome of considerable size and which he had exhibited at a previous meeting.

Mr. Wilson exhibited an upper lateral, which simulated in a remarkable degree a lower canine.

Mr. Macgregor showed two models, the one of a girl of 15, with a well marked V-shaped maxilla. The teeth were prominent, and he judged that the conformation of the mouth was due in a very great measure to the habit of food sucking of which he knew the patient was guilty.

The second model was of the upper jaw of a girl of 12, and peculiar in exhibiting a first right bicuspid of abnormal size and shape, from its appearance giving one the idea that it was probably a geminated tooth,—*Dental Record.*

